# The effects of inspiratory muscle training with pulmonary rehabilitation on NSCLC patients during radiation therapy: A pilot clinical study

**DOI:** 10.1111/1759-7714.14899

**Published:** 2023-04-20

**Authors:** Junghwa Do, Seung Hak Lee, Sang Ah Kim, A Hyun Kim, Ma. Nessa Gelvosa, Hwayeong Cheon, Jae Yong Jeon

**Affiliations:** ^1^ Department of Rehabilitation Medicine Asan Medical Center, University of Ulsan College of Medicine Seoul Republic of Korea; ^2^ Asan Institute for Life Sciences, Asan Medical Center, University of Ulsan College of Medicine Seoul Republic of Korea; ^3^ Biomedical Engineering Research Center, Asan Medical Center, University of Ulsan College of Medicine Seoul Republic of Korea

**Keywords:** exercise capacity, lung malignancy, rehabilitation, respiratory muscle strength

## Abstract

**Background:**

The effects of inspiratory muscle training (IMT) with pulmonary rehabilitation (PR) on patients with non‐small cell lung cancer (NSCLC) receiving radiotherapy (RT) have not previously been reported. This pilot study aimed to determine the effectiveness of IMT with PR on respiratory muscles and exercise capacity of NSCLC patients receiving RT.

**Methods:**

We retrospectively analyzed 20 patients who underwent RT for NSCLC. The rehabilitation included IMT, stretching, strengthening, and aerobic exercises three times a week for 4 weeks with concurrent RT. IMT training lasted 10 min, consisting of one cycle of 30 breaths using the Powerbreathe KH1 device in the hospital by a physical therapist. Patients underwent two IMT sessions at home daily at an intensity of approximately 30%–50% of the participant's maximum inspiratory muscle pressure (MIP) using the threshold IMT tool. We analyzed the results from the respiratory muscle strength test, pulmonary function test, 6‐min walk test (6MWT), cardiopulmonary function test, cycle endurance test (CET), Inbody test, grip measurement, knee extensor/flexor strength measurement, Cancer Core Quality of Life Questionnaire (EORTCQ‐C30), and NSCLC 13 (EORTC‐LC13).

**Results:**

There were no adverse events during evaluation and IMT with PR. MIP (60.1 ± 25.1 vs. 72.5 ± 31.9, *p* = 0.005), 6MWT (439.2 ± 97.1 vs. 60.7 ± 97.8, *p* = 0.002), CET (181.39 ± 193.12 vs. 123.6 ± 87.6, *p* = 0.001), knee extensor (14.4 ± 5.3 vs. 17.4 ± 5, *p* = 0.012), and knee flexor (14.0 ± 5.2 vs. 16.9 ± 5.5, *p* = 0.004) significantly improved after IMT with PR.

**Conclusion:**

IMT with PR appears effective on respiratory muscles and exercise capacity without adverse events in NSCLC patients who underwent RT.

## Graphical abstract

The effects of inspiratory muscle training (IMT) with pulmonary rehabilitation (PR) on patients with non‐small cell lung cancer (NSCLC) receiving radiotherapy (RT) have not been reported.

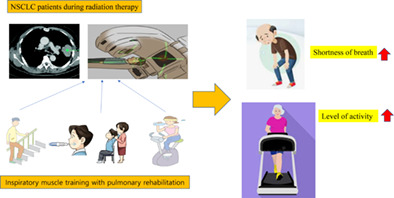

## INTRODUCTION

Radiotherapy (RT) of tumors located within or around the thoracic cavity unavoidably results in partial irradiation of the normal lung tissue.^1^ Radiation‐induced respiratory toxicity ranges from an often asymptomatic impairment of lung function to fibrosis and particularly radiation pneumonitis (RP), which lead to life‐threatening complications.^2^ Reduced pulmonary function was found to increase RP risk.^3^, ^4^, ^5^ A majority of lung cancer patients already have an impaired pulmonary function before RT.^2^ Physical and functional well‐being significantly decline, and esophagitis and fatigue are the most commonly reported during RT.^6^

Inspiratory muscle training (IMT) is a proposed form of prehabilitation^7^ and has recently become a popular and widely used training method.^8^ The rationale for IMT is that training at a higher level of inspiratory resistance may facilitate lung expansion in the postoperative period and help maintain patency of the smaller airways, thus resulting in enhanced diaphragm activity.^9^ Improving inspiratory muscle strength and endurance is a management strategy that may help relieve symptoms of dyspnea, thereby increasing the level of activity and improving the quality of life of patients with respiratory problems.

Methods of providing IMT include inspiratory threshold pressure loading, isocapnic/normocapnic hyperpnea, and inspiratory resistive flow loading.^10^ Respiratory muscle weakness has been suggested to contribute to the development of postoperative pulmonary complications, and reductions in both inspiratory and expiratory muscle strength have been demonstrated up to 12 weeks following thoracotomy in lung cancer.^11^

Preoperative IMT for at least 2 weeks before surgery with an intensity of 30%–60% of MIP has been shown to significantly improve respiratory muscle function in the early postoperative period following cardiothoracic or upper abdominal surgery, halving the risk of PPC. Molassiotis et al. showed that IMT for the management of breathlessness in patients with thoracic malignancies is not only feasible and acceptable for the patients but has positive effects on levels of breathlessness, anxiety, and fatigue.^12^

However, the effects of IMT on non‐small cell lung cancer (NSCLC) patients receiving RT have not been reported.

The purpose of this pilot study was to determine the effectiveness of IMT with PR on respiratory muscles and exercise capacity of patients with NSCLC receiving RT.

## METHODS

We conducted a retrospective review on patients who participated in a pulmonary rehabilitation (PR) program, including Powerbreathe, during RT. Data were collected from patients aged above 19 years old who received RT without comorbidities, including definitive, palliative, salvage, and postoperative RT, between January 2020 and December 2020 in our center. Patients with NSCLC stages I and IIIB, ECOG‐PS 0 to 2 were enrolled for the pulmonary program by a physician at the first visit. During this period, 24 patients underwent PR with IMT, and 16 completed whole sessions. We excluded patients who partially participated and completed only 12 sessions. Therefore, we included only patients who completed the entire rehabilitation program in our pilot study. During physical therapy, we performed the respiratory muscle strength test, pulmonary function test, 6‐min walk test (6MWT), cardiopulmonary function test, cycle endurance test, Inbody test, grip measurement, knee extensor/flexor strength measurement, Cancer Core Quality of Life Questionnaire (EORTCQ‐C30), and NSCLC 13 (EORTC‐LC13).

On the first day of the PR program, the respiratory muscle strength test, pulmonary function test, 6MWT, cardiopulmonary function test, cycle endurance test, Inbody test, grip measurement, knee extensor/flexor strength measurement, Cancer Core Quality of Life Questionnaire (EORTCQ‐C30), and NSCLC 13 (EORTC‐LC13) were evaluated and recorded for all patients. The program was performed simultaneously during the RT period. Each 60‐min exercise session was conducted under the supervision and guidance of a physical therapist. The training frequency was three sessions per week, resulting in a total of 12 training sessions.

The PR program included diaphragm breathing, stretching, aerobic exercise using cycling, treadmill walking, arm ergometry, and resistance training of arm and leg muscles. The exercises were controlled by physical therapists, and resistance strength and speed were adjusted based on the performance ability level. Patients performed endurance or interval trainings at moderate intensity. The patients were informed of the Borg scale of perceived exertion level and exercised at a intensity of 13–15 (somewhat hard to hard) on the Borg scale. The overall training intensity was increased gradually during the program. Peripheral capillary oxygen saturation were monitored during exercises using a portable oximeter. The exercises were stopped when desaturation occurred (oxygen saturation < 90%).

### Inspiratory muscle training

The total daily training time was 10 min, consisting of one cycle of 30 breaths using the Powerbreathe KH1 device (POWERbreatheKH1, HaB International Ltd) in the hospital by a physical therapist. As recommended by Reid et al.,^13^ the initial training intensity was set at 30% of the patient's maximum inspiratory pressure (PImax). The PImax percentage was increased as tolerated up to 5% weekly to a maximum, of 50% PImax. A Physical therapist provided Threshold IMT/PEP for home training and explained the methods of IMT training at home. Patients underwent two IMT sessions at home daily at an intensity equivalent to 30%–50% of the participant's maximum inspiratory muscle pressure (MIP) using the Threshold IMT tool. Participants were trained on how to use the device to complete sessions effectively. The durations of inspiration and expiration were 1.5–2 s and 6 s, respectively. During the training, the procedure was stopped if patients had any obvious discomfort, such as shortness of breath, dyspnea, or exhaustion.

### Assessments

We used the medical record of the respiratory muscle strength test, pulmonary function test, 6MWT, cardiopulmonary function test, cycle endurance test, In‐body test, grip measurement, knee extensor/flexor strength measurement, Cancer Core Quality of Life Questionnaire (EORTCQ‐C30), and NSCLC13 (EORTC‐LC13). Assessment were performed at baseline and after intervention as part of the routine clinical assessment in our center.

### Pulmonary function test

Forced vital capacity (FVC), forced expiratory volume in 1 s (FEV1), maximum inspiratory capacity (MIP), and maximum expiratory capacity (MEP) were measured with a digital spirometer (Pony FX, Cosmed Inc.). The measurements were taken with the patients seated upright in a chair with the nose blocked with a clip while slightly biting on the mouthpiece. This was implemented three times each and the maximum value for each was recorded.^14^

### Six‐minute walk test (6MWT)

The 6MWT was conducted in a 30‐m stretch corridor at the patient's own speed. Patients were instructed to walk as far as they could for 6 min and stop walking when exhausted. The modified Borg dyspnea scale was used to assess dyspnea before and after the test.^15^

### Cardiopulmonary function test

Cardiorespiratory function was measured using the cycle test (Ergoline 200 k; Ergoline GmbH). Patients commenced cycling at 20 W; this workload was increased by 25 W every minute. The test was completed when patients reached 85% of their estimated maximal heart rate. The cardiorespiratory test score was assessed as the power output that coincided with the 85% maximal heart rate.^16^

### Constant‐load exercise test (CET)

To assess a symptom‐limited maximal exercise endurance, a CET was performed with a cycle ergometer at a work rate equivalent to 75% of the previously achieved maximum resistance value. Patients were instructed to work the pedals (pedaling rate, 60/min) for as long as they could, and to stop pedaling when exhausted. The observer encouraged patients every 2 min and terminated the test after 15 min.^17^

### Inbody test

InBody S10 (Inbody Co.) was used to evaluate body composition. Eight‐point tetrapolar electrodes were applied; two electrodes were attached to each foot and hand at the four extremities of participants in a supine position.^18^

### Muscle strength test

The muscle strength of the knee extensor and flexor was measured using an Inbody dynamometer. The patient was instructed to exert maximum force for 3–5 s on sitting position. This was measured by isometricity. The test was performed three times and the maximum value was recorded.^13^

### Grip strength test

A digital dynamometer was used to measure grip strength. Each patient was seated with both feet flat on the floor, shoulders adducted, and the testing arm close to the body with the elbow fully extended. A physical therapist instructed them to produce their maximum grip strength, and the highest of three readings was recorded. Measurements were recorded for both the right and left hands.^19^

### Cancer core quality of life questionnaire (EORTCQ‐C13) and NSCLC13 (LC13)

The EORTC QLQ‐C30 is a self‐administered questionnaire consisting of 30 items that incorporate five functional scales (physical, functional, cognitive, emotional, and social performance), three symptom scales (fatigue, pain, and nausea and vomiting), and the scales of QoL and overall health status.^20^

### Statistical analysis

Statistical analyses were performed using SPSS version 18.0 software (SPSS Inc.). Continuous variables are expressed as mean ± standard deviation and categorical variables are expressed as numbers and percentages. Wilcoxon signed rank test was used to compare changes in the variables from baseline to the completion of the PR program. *p* < 0.05 was considered statistically significant.

## RESULTS

The average age of participants was 65.2 years. There were 15 men and five women. The average body mass index was 23.6. All patients were diagnosed with NSCLC, and 10, 55, and 25% of the patients had stage IIB, IIIA, and IIIB of the disease, respectively (Table 1). No adverse effects were observed during evaluation and IMT with PR. After the rehabilitation program, MIP (60.1 ± 25.1 vs. 72.5 ± 31.9, *p* = 0.005), 6MWT (439.2 ± 97.1 vs. 60.7 ± 97.8, *p* = 0.002), CET (181.39 ± 193.12 vs. 123.6 ± 87.6, *p* = 0.001), knee extensor, and knee flexor significantly improved. We found no statistically significant difference in the overall mean of EORTC C‐30, including global health status, functional scales (physical functioning, role functioning, emotional functioning, and cognitive functioning), symptom scales (fatigue, nausea and vomiting, pain, dyspnea, insomnia, appetite loss, constipation, diarrhea, and financial difficulties). Dysphagia symptoms in EORTC LC‐13 was significantly worsened at post‐evaluation. Other symptoms, such as dyspnea, coughing, peripheral neuropathy, and pain, were not significantly different after IMT with PR (Tables 2, 3, 4, 5).

**TABLE 1:** Baseline characteristics of patients

Characteristic	Subjects (*n* = 20)
Age (years)	65.2 ± 8.0
BMI (kg/m^2^)	23.6 ± 2.7
Male/female	15/5
NSCLC stage	
IB	1 (5%)
IIA	1 (5%)
IIB	2 (10%)
IIIA	11 (55%)
IIIB	5 (25%)

Abbreviations: BMI, body mass index; NSCLC, non‐small cell lung cancer.

**TABLE 2:** Pulmonary function during the initial evaluation and after the rehabilitation program

Variable	Initial evaluation (*n* = 20)	Post‐evaluation (*n* = 20)	*p*‐value
FVC (L)	2.7 ± 0.7	2.7 ± 0.7	0.179
FVC(% predicted value) (%)	87.1 ± 19.3	84.8 ± 18.0	0.107
FEV1 (L/s)	1.9 ± 0.5	1.9 ± 0.5	0.888
FEV1(% predicted value) (%)	84.0 ± 20.1	82.6 ± 19.0	0.751
MIP (cmH_2_O)	60.1 ± 25.1	72.5 ± 31.9	0.005
MIP (% predicted value) (%)	73.2 ± 26.8	87.0 ± 32.4	0.006
MEP (cmH_2_O)	57.3 ± 17.6	64.7 ± 18.4	0.070
MEP (% predicted value) (%)	52.6 ± 13.6	59.7 ± 17.0	0.067
PCF (lpm)	340.0 ± 96.1	352.0 ± 98.2	0.284

Abbreviations: FEV1, forced expiratory volume in 1 s; FVC, forced vital capacity; MEP, maximum expiratory capacity; MIP, maximum inspiratory capacity; PCF, peak cough flow; PFT, pulmonary function test.

**TABLE 3:** Physical evaluation during the initial evaluation and after the rehabilitation program

Variable	Initial evaluation (*n* = 20)	Post‐evaluation (*n* = 20)	*p*‐value
Vo2 max (VO_2_/kg max)	25.1 ± 6.7	24.7 ± 6.3	0.872
6 MWT (meter)	439.2 ± 97.1	460.7 ± 97.8	0.002*
Cycle ergometer test	181.39 ± 193.12	123.6 ± 87.6	0.001*
Max power (watt)	113.0 ± 38.6	113.2 ± 38.8	0.913
BMI (kg/m^2^)	23.6 ± 2.7	23.8 ± 2.7	0.879
Skeletal muscle (kg/m^2^)	27.5 ± 7.4	27.5 ± 6.9	0.573
Fat mass (kg/m^2^) (kg)	14.6 ± 5.6	14.8 ± 4.9	0.737
Knee extensor (rt) (kg)	29.5 ± 8.4	32.3 ± 8.9	0.008*
Knee extensor (lt) (kg)	27.1 ± 7.9	29.6 ± 7.9	0.008*
Knee flexor (rt) (kg)	14.4 ± 5.3	17.4 ± 5.5	0.012*
Knee flexor (rt) (kg)	14.0 ± 5.2	16.9 ± 5.5	0.004*
Grip strength (rt) (kg)	32.8 ± 9.5	32.5 ± 10.3	0.380
Grip strength (lt) (kg)	31.1 ± 8.9	31.2 ± 9.5	0.904

Abbreviations: 6MWT, 6‐min walk test; BMI, body mass index.

**TABLE 4:** EORTC C‐30 during the initial evaluation and after the rehabilitation program

Variable (scores)	Initial evaluation (*n* = 20)	Post‐evaluation (*n* = 20)	*p*‐value
Global health status/QOL	56.7 ± 24.6	60.4 ± 22.9	0.535
Functional scale			
Physical function	77.9 ± 14.5	76.3 ± 14.5	0.721
Role function	78.3 ± 18.8	74.1 ± 25.6	0.368
Emotional function	78.3 ± 18.8	79.5 ± 11.6	0.732
Cognitive function	76.6 ± 21.8	81.6 ± 17.0	0.326
Social function	74.9 ± 28.8	71.6 ± 27.6	0.710
Symptom scale			
Fatigue	36.1 ± 24.9	31.6 ± 23.1	0.163
Nausea and vomiting	18.3 ± 25.3	12.5 ± 19.4	0.151
Pain	22.5 ± 25.5	19.1 ± 14.5	0.590
Dyspnea	29.9 ± 21.3	33.3 ± 21.6	0.496
Insomnia	18.3 ± 27.5	21.6 ± 22.3	0.480
Appetite loss	20.3 ± 14.6	29.6 ± 35.9	0.777
Constipation	30.0 ± 35.7	28.3 ± 31.1	0.470
Diarrhea	21.6 ± 22.3	30.3 ± 35.7	0.089
Financial difficulties	28.3 ± 31.1	21.6 ± 29.1	0.891

Abbreviations: EORTC QLQ‐C30, The European Organization for Research and Treatment of Cancer Quality of Life Questionnaire Core 30.

**TABLE 5:** EORTC LC‐13 during the initial evaluation and after the rehabilitation program

Variable (scores)	Initial evaluation (*n* = 20)	Post‐evaluation (*n* = 20)	*p*‐value
Dyspnea	22.2 ± 16.1	27.7 ± 19.2	0.089
Coughing	21.6 ± 24.8	21.6 ± 19.5	0.891
Hemoptysis	4.9 ± 12.2	3.3 ± 10.2	0.317
Sore mouth	8.3 ± 14.8	13.3 ± 25.1	0.257
Dysphagia	8.3 ± 14.8	23.3 ± 21.8	0.008*
Peripheral neuropathy	13.3 ± 19.9	14.9 ± 22.8	0.796
Hair loss	26.6 ± 33.5	38.3 ± 36.3	0.322
Chest pain	18.3 ± 25.3	24.9 ± 28.3	0.236
Arm/shoulder pain	18.3 ± 27.5	9.9 ± 19.0	0.166
Other pain sites	19.9 ± 27.3	8.3 ± 14.8	0.070

Abbreviations: EORTC QLQ‐LC13, The European Organization for Research and Treatment of Cancer Quality of Life Questionnaire Lung Cancer 13.

## DISCUSSION

Reduced inspiratory muscle strength leads to limited vital capacity, shortening and weakness of the chest wall with associated poor ventilation, ineffective cough, and decreased exercise tolerance. We observed that a four‐week IMT with PR program improved respiratory muscle strength and exercise capacity in NSCLC patients during RT. This is of great importance because patient symptoms usually worsen as time goes by.

To our knowledge, this study is the first to report the effectiveness of IMT with PR on muscles and exercise capacity in patients with NSCLC receiving RT. Our study used various physical assessment methods to help understand NSCLC patients undergoing RT.

Many physical symptoms, such as dyspnea, fatigue, and pain, can appear during RT. RP or pulmonary inflammation due to RT can cause significant morbidity and occasionally mortality following thoracic RT.^21^ Clinical RP was experienced by 20% of NSCLC patients. The risk factors included poor performance status, low pulmonary function, comorbid lung disease, smoking history, and surgical resection.^4^ Therefore, improving performance status and exercise capacity is important, even during radiation treatment periods.

In our study, MIP significantly improved after IMT with PR. MEP and PCF did not significantly improve but improved at post‐evaluation. In previous randomized clinical trials (RCTs), inspiratory muscles and aerobic exercise training significantly facilitated respiratory muscle strength recovery, increased lung volume, and improved the distance covered in the 6MWT in NSCLC patients after video‐assisted thoracoscopic surgery (VATS). These improvements were observed as early as the second week and were sustained up to 12 weeks. This study included the early stage of NSCLC after VATS. In this study, MIP (60.1 ± 25.1 vs. 72.5 ± 31.9 cmH2O, *p* = 0.005) was lower compared to MIP (71.6 ± 34.9 vs. 94.3 ± 32.8 cmH2O, *p* = 0.018) in previous RCTs.^22^ Another study reported that additional IMT in patients at high risk of PPC significantly improved oxygenation up to 5 days after surgery when compared with standard physiotherapy alone. However, no differences in respiratory muscle strength or walked distance were detected between groups.^23^

In our study, there was no difference between FVC, FEV1, and baseline after 4 weeks. Improvements in the physiological index of lung capacity may be difficult to attain in NSCLC. In addition, there was no improvement in dyspnea symptoms in EORTC C30 and LC13. This was different from the results reported in another RCT that IMT training is effective against dyspnea and breathlessness in patients with thoracic malignancies. This may be the effect during RT, which may cause various physical symptoms. Symptoms such as appetite loss, dysphagia, and chest pain did not change significantly after 4 weeks but tended to increase. In addition, the duration and intensity of training was different. In this study, the protocol comprised five IMT sessions weekly for 12 weeks for a total of 30 min/day. The initial training was set as 40% and progressed to a maximum of 70% PImax compared our protocol which progressed from 30% PImax to 50% PImax.

Our program was ineffective as regards the breathlessness symptom; however, it was effective in improving inspiratory muscle and exercise tolerance. The 6MWT, CET, and strength of lower extremities significantly improved after IMT with PR. There have been previous studies on rehabilitation in patients with NSCLC receiving RT. Simultaneous PR improved pulmonary function, particularly in measures of FEV1, and exercise capacity in patients with lung or esophageal cancer even after radiotherapy treatment.^24^ Another preliminary study demonstrated that PR programs improved exercise tolerance as measured by the 6MWT among inpatients receiving concurrent chemoradiotherapy.^25^

There were several limitations to this study. First, it was a retrospective pilot study with a small sample size and the power analysis could not be stated. There is currently not enough evidence of PR on patients receiving RT and PR is not commonly prescribed. There was also no control group and the absence of a control group prevents the additional benefit of IMT from being demonstrated. Therefore, the value of these results in clinical practice may be limited. Based on the results of this preliminary pilot study, prospective RCTs should be performed to provide more supportive evidence.

In conclusion, inspiratory muscle training with PR appears to be effective on the respiratory muscles and exercise capacity without adverse events in NSCLC patients who underwent RT.

## AUTHOR CONTRIBUTION

All authors have contributed significantly, and that all authors are in agreement with the content of the manuscript: Conception/Design: Junghwa Do, Jae Yong Jeon; Collection and/or assembly of data: Junghwa Do, Sang Ah Kim, A hyun kim; Data analysis and interpretation: all authors; Manuscript writing: all authors; Final approval of manuscript: all authors.

## CONFLICT OF INTEREST STATEMENT

The authors declare that they have no conflicts of interest.
